# Cocoa pulp in beer production: Applicability and fermentative process performance

**DOI:** 10.1371/journal.pone.0175677

**Published:** 2017-04-18

**Authors:** Cassiane da Silva Oliveira Nunes, Giovani Brandão Mafra de Carvalho, Marília Lordêlo Cardoso da Silva, Gervásio Paulo da Silva, Bruna Aparecida Souza Machado, Ana Paula Trovatti Uetanabaro

**Affiliations:** 1 Department of Biology and Biotechnology of Microorganisms, State University of Santa Cruz, Ilhéus, Bahia, Brazil; 2 Bahia Federal Institute Catu Campus, Catu, Bahia, Brazil; 3 Department of Biotechnology, State University of Feira de Santana, Feira de Santana, Bahia, Brazil; 4 Department of Education, State University of Bahia, Senhor do Bonfim, Bahia, Brazil; 5 Department of Biotechnology and Food, Faculty of Technology, SENAI/CIMATEC, National Service of Industrial Learning–SENAI, Salvador, Bahia, Brazil; 6 Institute of Technology in Health, Faculty of Technology, SENAI/CIMATEC, National Service of Industrial Learning–SENAI, Salvador, Bahia, Brazil; College of Agricultural Sciences, UNITED STATES

## Abstract

This work evaluated the effect of cocoa pulp as a malt adjunct on the parameters of fermentation for beer production on a pilot scale. For this purpose, yeast isolated from the spontaneous fermentation of cachaça (SC52), belonging to the strain bank of the State University of Feira de Santana-Ba (Brazil), and a commercial strain of ale yeast (Safale S-04 Belgium) were used. The beer produced was subjected to acceptance and purchase intention tests for sensorial analysis. At the beginning of fermentation, 30% cocoa pulp (adjunct) was added to the wort at 12°P concentration. The production of beer on a pilot scale was carried out in a bioreactor with a 100-liter capacity, a usable volume of 60 liters, a temperature of 22°C and a fermentation time of 96 hours. The fermentation parameters evaluated were consumption of fermentable sugars and production of ethanol, glycerol and esters. The beer produced using the adjunct and yeast SC52 showed better fermentation performance and better acceptance according to sensorial analysis.

## Introduction

The need to produce quality beer in a short time frame has led breweries to search for new alternatives, such as the use of adjuncts, production of high-gravity beer, and utilization of selected yeasts. Beer is traditionally produced by fermentation of the brewing wort with an original extract of 12°P (°P is the weight of extract (sugar) equivalent to the weight of sucrose in a 100 g solution at 20°C), with a final ethanol content of 5% (v/v). Malt, hops, water and yeasts are used in beer production, and adjuncts can also be added. A 12°P wort contains approximately 90 g/liter of fermentable sugars (maltose, maltotriose and especially glucose) as well as non-fermentable sugars (especially dextrin) [1–3].

In fermentation, a balance between yeast growth and metabolism should be achieved, such that the desirable compounds are produced within the time frame required for the process. Furthermore, the parameters influencing the process, such as nutrient availability, correct inoculation (pitching), dissolved oxygen concentration and temperature control, all affect the growth of the yeast cells [4–6]. A relevant consideration in the current beer market is the use of adjuncts that have a high concentration of fermentable sugars, which contribute to a higher production of ethanol from each °Plato of extract [2,7–8].

Adjuncts are particularly used as a cheaper wort than that obtained using only malt. Beer obtained using an adjunct also has other advantages, such as a brighter color and greater physical stability in addition to increased production and a final product with special characteristics [9]. Greater physical stability occurs because the adjuncts contribute very little to the protein material for the wort and beer, an advantage in terms of colloidal stability [10]. Additionally, the use of adjuncts can lead to alterations in beer characteristics, for example, a modified aroma profile.

According to Saerens et al. [11] and Hiralal et al. [12], the composition of the wort, the fermentation conditions, the proportion of each ester produced individually and the type of yeast used all influence the flavor profile of beer. The flavor of beer also depends on the balance between compounds such as acids, alcohols, aldehydes, ketones and esters. The esters considered to be the most important active flavors in beer are acetate esters (ethyl acetate, isoamyl acetate, isobutyl acetate, phenyl ethyl acetate) and ethylic esters (caproate and ethyl caprylate) [13–15].

In addition to providing a beer with a characteristic aroma, adjuncts should be chosen by regional availability. Within this context, the application of cocoa pulp from southern Bahia is an adjunct option for beer production due to such characteristics as the appropriate quantity of fermentable sugars and wide availability. Additionally, cocoa pulp has important nutrients for the fermentable medium, including polyphenols and minerals [16]. Thus, the use of cocoa pulp as an adjunct for beer production can provide an alcoholic beverage with distinct chemical and sensorial characteristics.

Cocoa (*Theobroma cacao* L.), a native of the tropical forests of South America, is a member of the Malvaceae family and is known worldwide for the use of its seeds in chocolate production [17]. Cocoa pulp is a substrate rich in nutrients and can be utilized in industrial processes to produce various sub-products [18–19]. Indeed, its use as a raw material is advantageous because, compared with other potentially useful tropical fruits, cocoa is an abundant product derived from an already established culture. Many studies have investigated utilizing the pulp, which confers special and attractive flavors, to produce juices, jams, compotes, fermented beverages and other processed products [16].

The objectives of this study were to evaluate the performance of beer fermentation on a pilot scale using cocoa pulp as an adjunct and strains of *Saccharomyces cerevisiae* isolated from the spontaneous fermentation of cachaça (SC52) and commercial ale (S-04) and to assess the beverage’s acceptance through a sensorial test.

## Materials and methods

### Beer production and fermentation conditions

The wort used was produced at the Laboratory of Fermentation of the State University of Feira de Santana–Ba (Brazil). To decrease the grain size and facilitate hydrolysis catalyzed by enzymes during wort production, 8.80 kg of malt was milled (*Chateau* Pilsen 3.25 EBC, origin Belgium) using a bench mill, and the milled malt was mixed with water at 35°C. Wort production was conducted in a 60-L recipient with manual agitation, LPG gas heating, with the time and temperature controlled using a chronometer and a digital thermometer.

The temperature varied from 35 to 76°C. The initial pH was adjusted to 5.4 by adding lactic acid and was adjusted with CaCl_2_ at the rate of 1.26 g/kg of malt. The wort production ramp used was adapted from Carvalho and Zambiazi [20]. At the end of wort production at 72°C, the saccharification of the malt starch was evaluated using an iodine solution at 0.2 N. After confirmation of complete starch hydrolysis, the solution was heated to 76°C to inactivate the enzymes present, filtered and transferred to the 60-L gas-heated boiling recipient. The wort was then boiled for one hour. At the beginning of boiling, hop extract was added at 0.10 g/L concentration. The wort was kept at the boiling temperature for 60 min, after which the hops were added in pellets at 0.82 g/L concentration relative to the initial volume.

The cocoa pulp was obtained from the fruits of clone VB 1151, provided by the Mars Center for Cocoa Science, which is harvested at optimum ripeness for consumption because this is not a climatic fruit. To avoid compromising the quality of the final pulp, the selection step consisted of the rejection of defective fruits as well as choosing commercial fruits based on size and color. The selected fruits were washed with clean tap water to remove impurities, sanitized for 30 minutes in 100 mg.mL^-1^ free chlorine solution, and rinsed with clean tap water to remove excess free chlorine. After that, the cocoa fruits were broken, pulped using a bench depulper (model DM-Ji-05, Macanuda), fractionated and stored in flexible polyethylene bags. The pulp fractions were then frozen and stored at -18°C. The pulp was used as an adjunct for the 12°P wort, at a concentration of 30%, which in previous experiments provided the optimal contribution for the fermentative performance of the evaluated yeasts (data not shown). The composition of the wort is described in Table 1. The composition of the obtained pulp, determined according to Dias et al. [16], was 18% total sugars, 10% reducing agents, 1.07% starch and 0.5% total pectin.

**Table 1:** Must properties with 30% cocoa pulp added.

	12°P
	(Means ± standard deviation)*
Glucose (g/L)	23.12±0.20
Fructose (g/L)	14.10±0.30
Maltose (g/L)	45.20±0.28
Maltotriose (g/L)	12.30±0.15
pH	5.30±0.01
European bitterness units (EBU)	25.0±0.02

*Means three replicates.

Before being added to the brewing wort as an adjunct, the cocoa pulp was subjected to enzymatic treatment to reduce viscosity. The enzyme used was polygalacturonase (7900 PGNU/mL, 55°C for one hour, pH 3.6) obtained from *Aspergillus niger* and *Aspergillus aculeatus* (Pectinex Ultra Clear, Novozymes). The pulp was added to the wort after the boiling phase, at the beginning of fermentation, according to Carvalho et al. [21]. The pilot-scale fermentation was performed in a 100-L capacity conical cylinder bioreactor with a useful volume of 60 L, a constant temperature of 22°C and a 12°P wort concentration.

This fermentation was followed with daily measurements of the apparent extract consumption and ethanol production at regular intervals until the apparent attenuation reached approximately 70–75%. After reaching the end of fermentation, the beer, considered green, was matured at 12°C for 14 days plus 2 days at 0°C to improve clarification. After maturation, the samples were frozen in an ultra-freezer at -80°C for analysis of esters (ethyl acetate, isoamyl acetate, isobutyl acetate, phenyl ethyl acetate, ethyl caproate and ethyl caprylate).

### Yeast strains

The *S*. *cerevisiae* strains used in this work were SC52, isolated from the spontaneous fermentation of cachaça and deposited in the culture collection of the State University of Feira de Santana (UEFS), Bahia (Brazil), and commercial ale (Safale S-04, Belgium). The SC52 strain was selected for its good performance in the fermentative process in a previous study performed with pure malt wort (data not shown).

The stock culture of SC52 was maintained at 4°C in a slant of Sabouraud agar. A pure culture was propagated to obtain sufficient biomass to start fermentation at 1–2 × 10^7^ cells/mL. Plugs were cut from the Sabouraud agar stock culture and incubated at 30°C for 24 hours. The plugs were then transferred for propagation to a 1000-mL Erlenmeyer flask containing 800 mL of pure 12°P malt wort via superficial scraping of the medium using a platinum flap under aseptic conditions. The flask was incubated at 30°C with rotation at 150 rpm for 24 hours. To initiate fermentation, the commercial yeast was weighed as per the manufacturer’s specifications and propagated in pure malt wort.

### Determination of the cell concentration

The concentration of cells in suspension was determined using a Neubauer chamber, and the result is expressed in cells/mL. Cell viability was determined by the International Method of Coloration using methyl blue according to the American Society of Brewing Chemists (ASBC) [22].

### Wort and beer analyses

The apparent extract of the wort and beer was determined using a bench densitometer (Rudolph Research Analytical, Tecnal), and the results are expressed in °Plato. The pH was determined using a digital pH meter (PH-1700; Instrutherm). The bitterness was analyzed using a spectrophotometer (Evolution 220, Thermo Scientific) at a wavelength of 275 nm. The color of the beer was also analyzed using a spectrophotometer (Spectrophotometer UV mini 1240, Shimadzu) at a wavelength of 430 nm, with water as the blank [23]. The analyses were performed in triplicate, and all of the results are shown as means ± standard deviation.

During fermentation, samples were removed at regular intervals, placed into Eppendorf tubes and analyzed as described below. Prior to the analysis, the beer samples were degassed by vigorous agitation of the placing Eppendorf tubes for one minute followed by centrifuged at 4000 rpm for 10 minutes. The supernatant was used to follow and quantify fermentation compounds: alcohols (ethanol, glycerol and methanol), carbohydrates (glucose, fructose, maltose and maltotriose), organic acids (citric acid, ascetic acid, lactic acid, succinic acid and formic acid) and esters (ethyl acetate, isoamyl acetate, isobutyl acetate, phenyl ethyl acetate, ethyl caproate and ethyl caprylate).

### Analysis of carbohydrates, alcohols and organic acids by high-performance liquid chromatography (HPLC)

To quantify fermentation compounds by HPLC, samples were diluted 10 times in ultrapure water and filtered through 0.45-μm-pore and 13-mm-diameter polyvinylidene difluoride membranes (Millex HV, Millipore). Standard deviations were determined for metabolites (glucose, fructose, maltose, maltotriose, ethanol, methanol, glycerol, citric, aseptic, lactic, succinic and formic acids) (Sigma-Aldrich) diluted in ultrapure water (Direct Q3UV, Millipore, Massachusetts, USA). An Ultimate 3000 (Dionex, Germany) equipped with a UV-Vis detector (using a wavelength of 210 nm) for detecting organic acids and the refraction index of sugars and alcohols (Sodhex RI-101, Showa Denko, Japan) was used.

To separate compounds, a Rezex ROA Organic Acids ionic exchange column (300 × 7.8 mm; Phenomenex, Torrance, CA, USA) was used at 60°C, with 0.005 M sulfuric acid as the mobile phase with a flow rate of 0.6 mL.min^-1^. The chromatograms were integrated using the software Chromeleon Server Monitor (Dionex); identification was performed by comparison of the retention times and quantification according to the standard deviation for each analyte.

### Calculation of fermentation parameters

#### Specific rate

The specific rate of substrate consumption (μSi) was defined as μSi = (1/X) × dSi/dt, where Si represents the type of carbohydrate analyzed (glucose, fructose, maltose and maltotriose). The specific rate of product formation (μP) was defined as μP = (1/X) × dP/dt, where P represents the product formed (ethanol). The derivatives dSi/dt and dP/dt were calculated according to the method proposed by Le Duy and Zajic [24].

#### Volumetric productivity (Qp) and yield (Yp/s) in ethanol

The volumetric productivity in ethanol (rate between the ethanol produced and total time of fermentation, g/L.h) and yield coefficient in ethanol (rate between the ethanol produced and the extract consumed, g/g) were determined after 96 hours of fermentation.

#### Apparent degree of fermentation (%)

The apparent degree of fermentation of the wort—that is, the proportion of extracts (dissolved solids) that can be fermented—is calculated using the following formula [1]: fermentability (%) = [(initial apparent extract–final apparent extract) / (initial apparent extract)] × 100.

### Analysis of esters by solid-phase microextraction (SPME)

Samples and standards: The standards were ethyl acetate, isoamyl acetate, isobutyl acetate, phenyl ethyl acetate, ethyl caproate and ethyl caprylate acquired from Sigma-Aldrich at >98% purity.

Preparation of standards and samples: A stock solution of the standards was prepared by dissolving the compounds in 96% ethanol and storing at 4°C. To prepare standard solutions, 9.5 mL of deionized water was added to a 20-mL vial, followed by the addition of 30 μL of 1-pentanol (5 mg/ml) as an internal standard and 25 μl of the stock solution, resulting in the following concentrations: ethyl acetate, 7.37 mg/L; isobutyl acetate, 1.08 mg/L; isoamyl acetate, 7.39 mg/L; ethyl caproate, 1.37 mg/L; ethyl caprylate, 2.02 mg/L; and 2-phenyl-ethyl acetate, 7.0 mg/L. The vial was sealed for the analysis with a final volume of 10 mL. Before the analysis, the beer samples were transferred to a 100-mL Erlenmeyer flask and agitated for 10 seconds; 9.970 mL of the sample and 30 μL of the internal standard were transferred to the vial. The samples were then analyzed by SPME.

SPME Headspace Analysis: A support consisting of the SPME Supelco (manual) fiber and a divinylbenzene/carboxin/polydimethylsiloxane fiber (DVB/CAR/PDMS) of 50/30 μM was used for SPME. Before use, the fiber was pre-conditioned for 2 hours in the injector of the gas chromatograph (CG) at 300°C. The SPME fiber was desorbed for 5 minutes. The samples were heated in a water bath at 50°C for 60 minutes to start the analysis.

### Sensorial analysis: Acceptance test

Acceptability testing is a method for evaluating a consumer product in a blind manner that refers to like or dislike responses collected on a graded scale. The industry standard for measuring acceptability is the nine-point hedonic scale, which consists of four phrases for like and four for dislike, with the modifiers slightly, moderately, very much, and extremely. The beers produced with the cocoa pulp as an adjunct at 30% concentration using the yeasts SC52 and ale S-04 were assessed. The samples were served at 8°C in 100-mL glasses. A glass of water at room temperature was served with the samples for mouth rinsing. The test to evaluate the attributes of taste, aroma and overall impression was performed under laboratory conditions. Each sample was analyzed individually by 90 non-trained tasters who were requested to attribute a mark using the 9-point hedonic scale, with 1 being “extremely dislike” and 9 being “extremely like” (S1 Fig). The intention to purchase the product was also evaluated on the same form [25]. The sensorial test was previously approved by the Ethics Research Committee of the State University of Santa Cruz, Ilhéus, Bahia (CEP/UESC) and ruling number 1.315.793. The results were analyzed by an analysis of variance (ANOVA) F test using the statistical software SISVAR 5.0.

## Results

### Influence of cocoa pulp as an adjunct on fermentation performance

An evaluation of beer production on a pilot scale using cocoa pulp as an adjunct at 30% concentration, the SC52 strain and the commercial strain S-04 at 22°C and 12°P wort original extract concentration was performed. The addition of cocoa pulp as an adjunct was intended to increase the concentration of the main products of fermentation and to produce a beer with distinct organoleptic characteristics.

**Fig 1:**
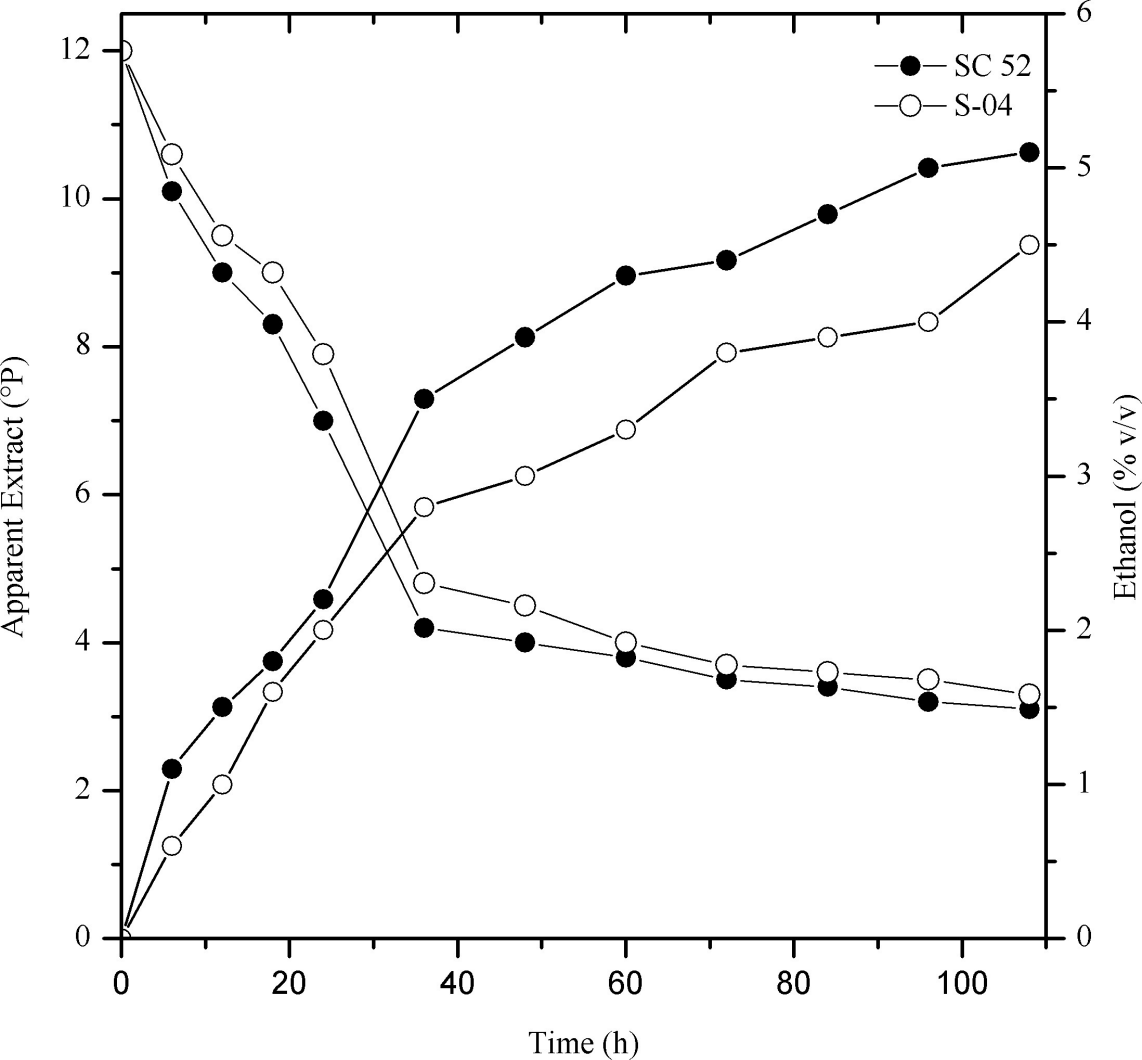
Consumption of apparent extract (°P) and the production of ethanol % (v/v) by the yeasts SC52 (isolated from spontaneous fermentation) and S-04 (commercial) using 12°P all-malt wort at 22°C.

According to Piddocke et al. [26], the main products of beer fermentation are ethanol, carbon dioxide, glycerol, and yeast biomass. The performance of the strain SC52 throughout fermentation using cocoa pulp as a malt adjunct was evaluated based on the consumption of total sugars (apparent extract, expressed in °Plato - °P) and fermentable sugars (glucose, fructose, maltose and maltotriose), the production of ethanol, glycerol and methanol, the volumetric productivity in ethanol and the yield compared with that of the commercial yeast S-04. In a previous study performed on a laboratory scale using the yeast strains SC52 and S-04 and all-malt wort, it was observed that SC52 resulted in higher consumption of extract and ethanol production than S-04, as shown in Fig 1.

**Fig 2:**
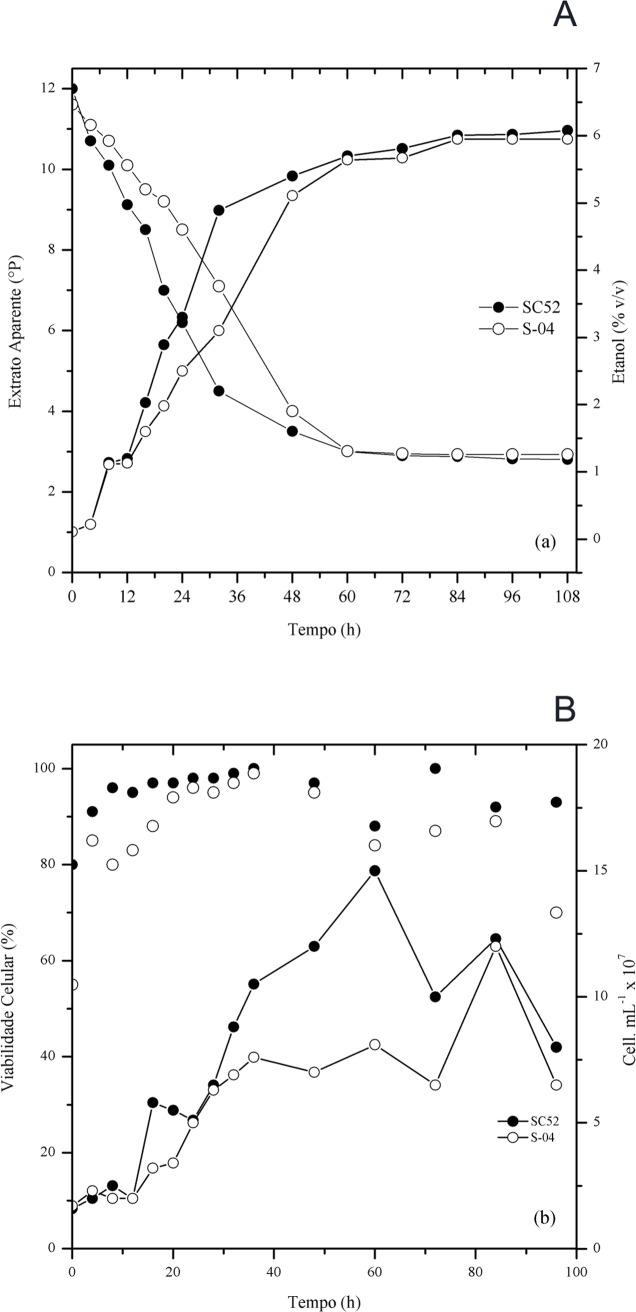
Apparent extract (°P), production of ethanol % (v/v) (a), cell viability (%), and cell concentration (cells/mL) (b) of the SC52 and S-04 yeast strains.

Regarding the pilot-scale production using cocoa pulp, it was observed that the specific rate of SC52 fermentation during the first 32 hours was higher than that of S-04, reaching an apparent degree of fermentation of 76% in 72 hours; compared with the S-04 strain, 84 hours was necessary to reach 75%. The consumption of substrate (apparent extract °P) was intimately linked to the increase in suspended yeast cells (Fig 2). Regarding cellular growth, the viability percentage of SC52 was higher, approximately 80%, at the beginning of fermentation.

Despite this difference at the beginning of fermentation, after 24 hours, the viability percentage was approximately 90% for both strains. In this interval (0 to 32 h), the apparent extract decreased approximately by 62% for the SC52 strain and 41% for the S-04 strain, reaching values of 4.5°P and 7.1°P, respectively. In this period, the production of ethanol was 4.89% and 3.1% (v/v) for SC52 and S04, respectively. The final concentration of ethanol was higher for the SC52 strain than for the S-04 strain, consequently with a lower apparent extract. After 96 hours, the final concentration of ethanol reached a percentage of 6.02% (v/v) for SC52 and 5.95% (v/v) for S-04.

**Fig 3:**
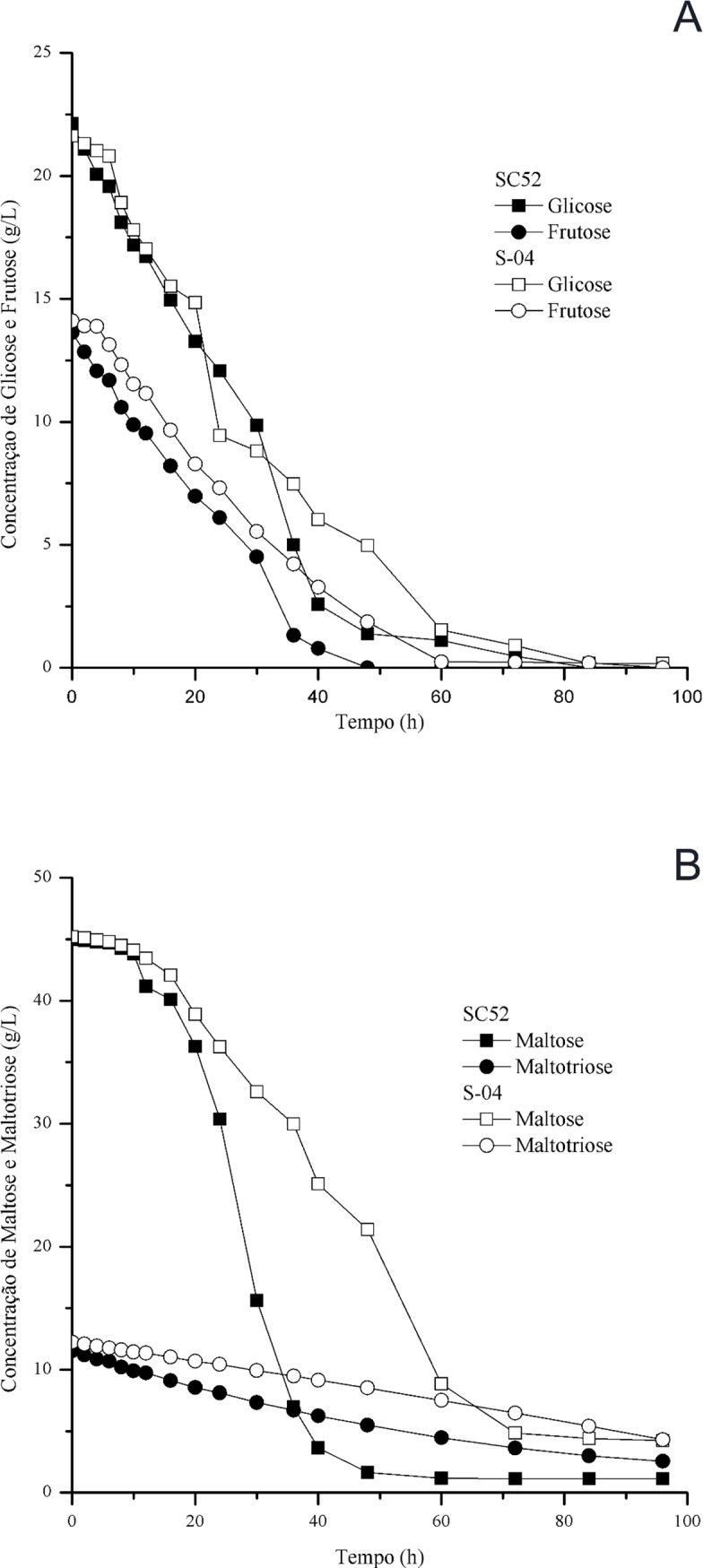
Concentration of fermentable sugars, glucose, fructose (a) and maltose, maltotriose (b), of the SC52 and S-04 yeast strains.

Compared with the fermentation in all-malt wort, the cocoa pulp contributed to an increase in ethanol production by the yeasts (Fig 2). Fermentable sugars were consumed in the following order: glucose, fructose, maltose and maltotriose (Fig 3). The final concentrations of glucose and fructose were similar for both beers. However, for maltose and maltotriose, the highest residual concentration was found with fermentation using the commercial yeast. The SC52 strain showed a higher efficiency in the consumption of sugars than the commercial yeast, justifying the higher observed volumetric productivity (0.50 x 0.48 g/L.h) and yield (0.46 x 0.44 g/g), respectively.

Data concerning fermentation are listed in Table 2. The final pH of the beer produced by the SC52 yeast strain was higher than that produced by the S-04 strain, likely due to a lower production of organic acids such as citric and succinic acids. Other acids, that is, acetic, lactic and formic acids, were not detected in the final product. Regarding color, the beer produced with the commercial yeast S-04 was lighter, at 8.0 EBC; however, the color was 9.0 EBC using the SC52 strain. The bitterness values were 16.4 and 15.8 BU for the SC52 and S-04 strains, respectively, with the less bitter beer being that produced using the commercial yeast.

**Table 2:** Data for fermentation on a pilot scale using an adjunct. The data were analyzed in triplicate, and the results are expressed as the mean ± standard deviation.

	SC52	S-04
Fermentation time (days)	4.0	4.0
Final Apparent Extract (° P)	2.8^a^±0.00	2.95^b^±0.01
Concentration of Residual Glucose (g / L)	0.0 ^a^	0.17 ^b^ ±0.01
Concentration of Residual Fructose (g / L)	0.0	0.0±0.01
Concentration of Residual Maltose (g / L)	1.12^a^ ±0.2	4.24^b^ ±0.3
Concentration of Residual Maltotriose (g / L)	2.56^a^ ±0.3	4.31^b^ ±0.2
Concentration of ethanol% (v/v)	6.02^a^ ±0.15	5.95^b^ ±0.2
Beer pH	4.3^a^ ±0.01	4.1^b^ ±0.01
Color (EBC)	9.0^a^ ±0.02	8.0^b^ ±0.01
Bitterness (EBU)	16.4^a^ ±0.01	14.8^b^ ±0.01
Citric Acid (g/L)	2.29^a^ ±0.25	2.89^b^ ±0.3
Succinic Acid (g/L)	1.44^a^ ±0.31	1.98^b^ ±0.18

Means marked with the same letter between rows are not significantly different (p>0.05) by the Tukey test.

**Fig 4:**
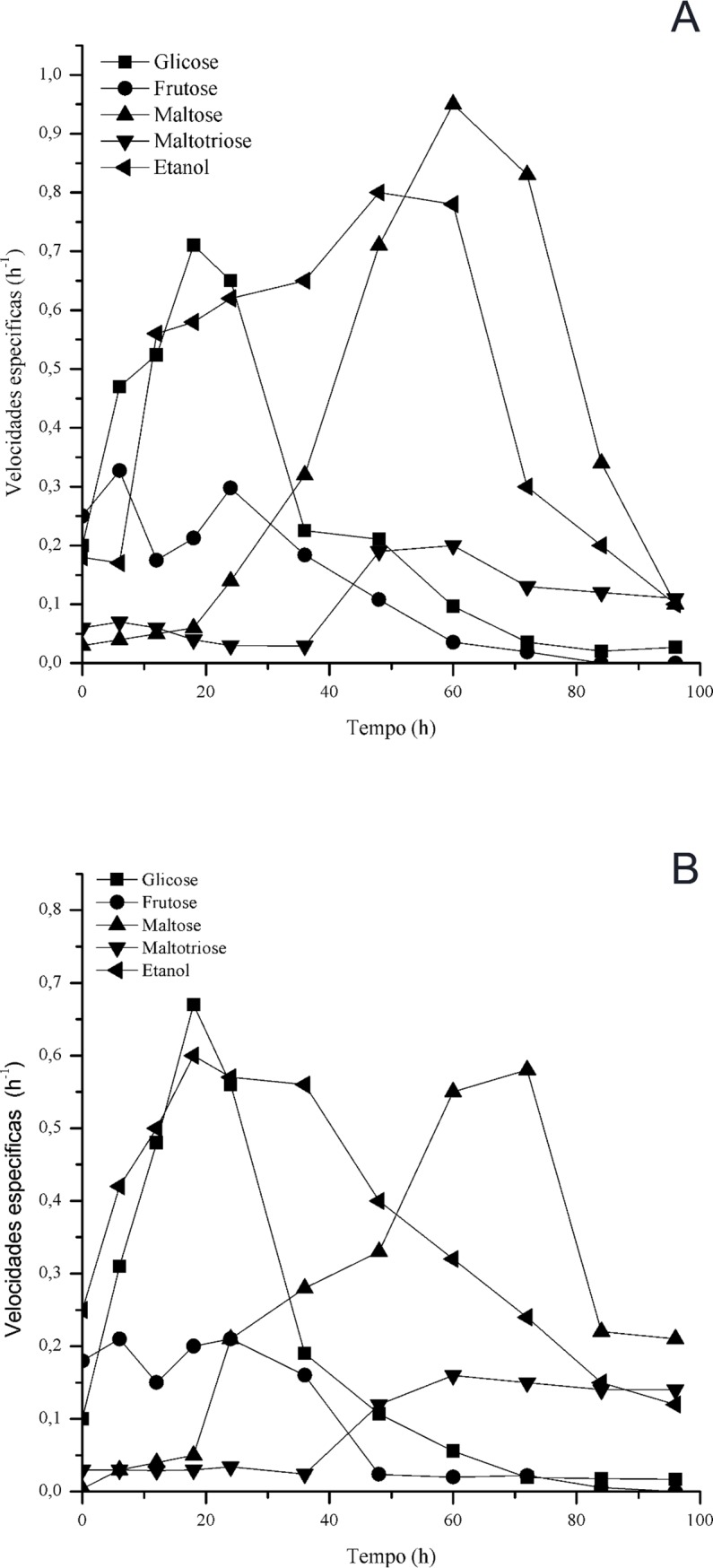
Specific rate of sugar consumption and formation of ethanol by the yeasts SC52 (a) and S-04 (b).

**Fig 5:**
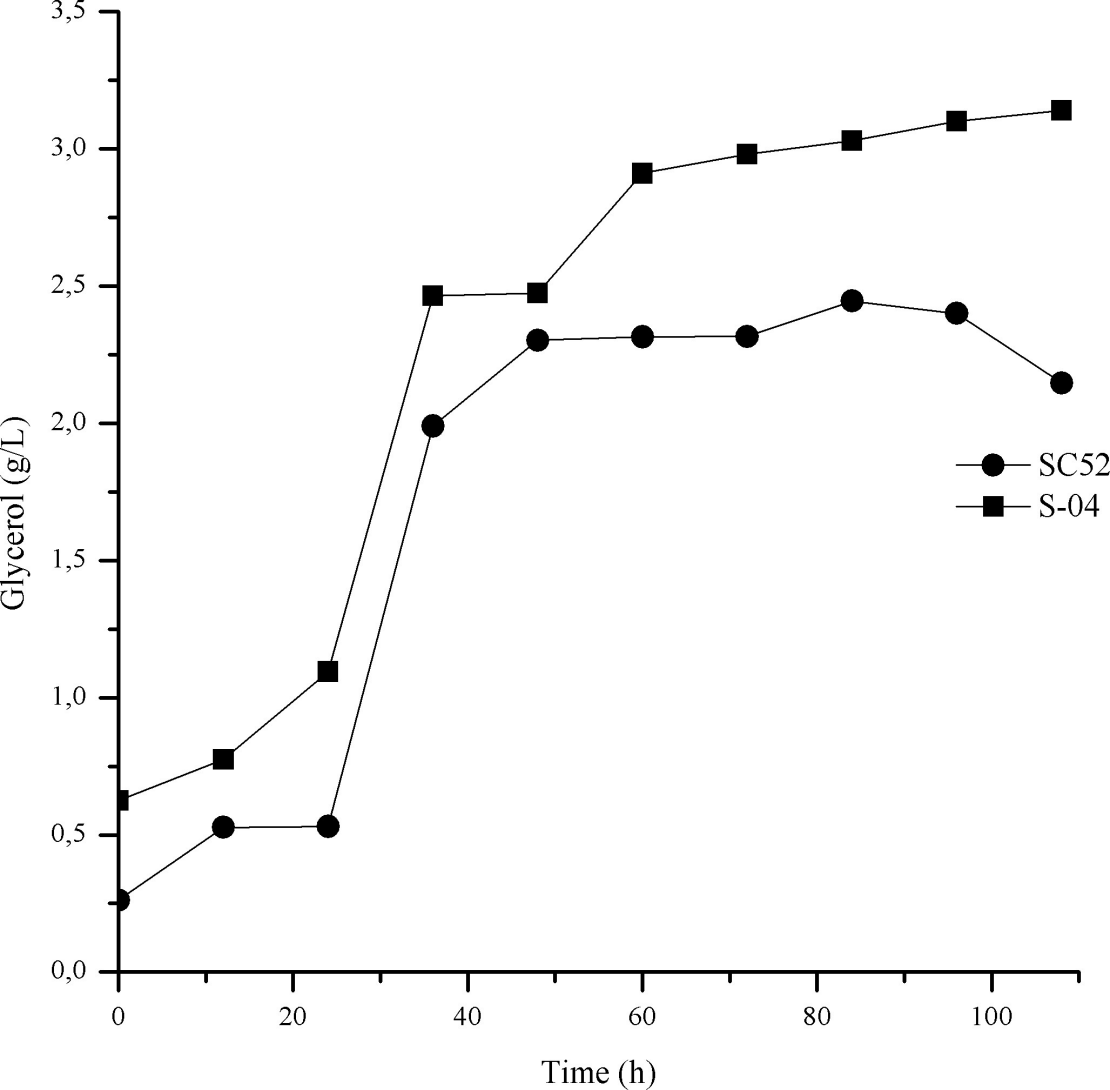
Production of glycerol during fermentation of wort containing cocoa pulp by strains SC52 and S-04.

The specific rate of sugar consumption was higher for SC52 than for S-04 (Fig 4). The specific rate of sugar consumption and production of ethanol had similar profiles and correlated with the type of alcoholic fermentation. This configuration represents the case in which the ethanol produced is directly linked to the reaction of sugar consumption—in this case, glucose, fructose, maltose and maltotriose. Regarding the production of glycerol, it was observed that the beer produced with S52 had a lower concentration throughout fermentation than that produced with S-04. At the end of fermentation, the maximum values reached were 2.4 and 3.1 g/L for SC52 and S-04, respectively (Fig 5). With regard to the production of methanol, which is a toxic compound, its presence was not observed in any beer produced using cocoa pulp as an adjunct.

### Concentration of esters in beers

The volatile esters produced during fermentation were quantified and analyzed by SPME, and the results are described in Table 3. The wort containing cocoa pulp (30%) and yeast strain SC52 resulted in an increase in the concentration of the studied acetate and ethyl esters compared with fermentation using the commercial yeast S-04. The esters present in higher quantity were ethyl caprylate, phenyl ethyl acetate and isoamyl acetate, in that order. An increase of 132% for acetate esters and 69% for ethyl esters was observed with fermentation using the yeast SC52.

**Table 3:** Concentration of esters (mg/L) and their respective thresholds [27–28] in beers produced using the strains SC52 and S-04.

	SC52	S-04	Value *Threshold*	*Flavor*
Ethyl acetate	2.03^a^ ± 0.01	1.30^b^ ± 0.017	21–30	Solvent
Isobutyl acetate	1.39^a^ ± 0.01	0.59^b^ ± 0.01	1.6	Fruitful
Isoamyl acetate	40.73^a^ ± 0.01	17.73^b^ ± 0.01	1.4	Banana
Ethyl caproate	19.86^a^ ± 0.01	14.57^b^ ± 0.01	0.21	Green apple
Ethyl caprylate	224.29 ^a^± 0.01	129.69^b^ ± 0.01	0.9	Green apple
Phenyl ethyl acetate	53.91^a^ ± 0.01	22.71^b^ ± 0.01	3.8	Roses, honey

Data were analyzed in triplicate, and the results are expressed as the mean ± standard deviation. Means marked with the same letter between rows are not significantly different (p>0.05) by the Tukey test.

In the beer produced with SC52, isoamyl acetate and phenyl ethyl increased 130% and 137%, respectively, in relation to that produced with the commercial yeast S-04. With both yeast strains, ethyl acetate, which is responsible for the solvent aroma, was produced in a quantity below the level of perception. When using SC52, isobutyl acetate was 134% higher and ethyl caproate and ethyl caprylate 36% and 73% higher, respectively.

### Sensorial analysis of the beverage

After maturation, the beers were subjected to sensorial analysis to evaluate acceptance among consumers. The samples were analyzed by 90 beer tasters, aged older than 20 years old, of whom 62 were male and 28 female. Three sensorial parameters were assessed to evaluate the acceptability of the beers: aroma, taste and overall impression. Table 4 shows the grades given to each sample by the non-qualified tasters using the nine-point hedonic scale.

**Table 4:** Averages of consumer acceptance in relation to aroma, taste and overall impression of beer samples produced with the yeasts SC52 and S-04.

	Averages Obtained	
Attributes	Beer produced with 30% cocoa pulp and strain SC52	Beer produced with 30% cocoa pulp and strain S-04
Aroma	8.10^a^ ± 0.22	7.81^b^ ± 0.22
Taste	7.63 ^a^ ± 0.22	6.88 ^b^ ± 0.22
Overall impression	8.10^a^ ± 0.22	7.16^b^ ± 0.22

The same letters within the same line indicate that there was no significant difference between the samples, p>0.05.

**Fig 6:**
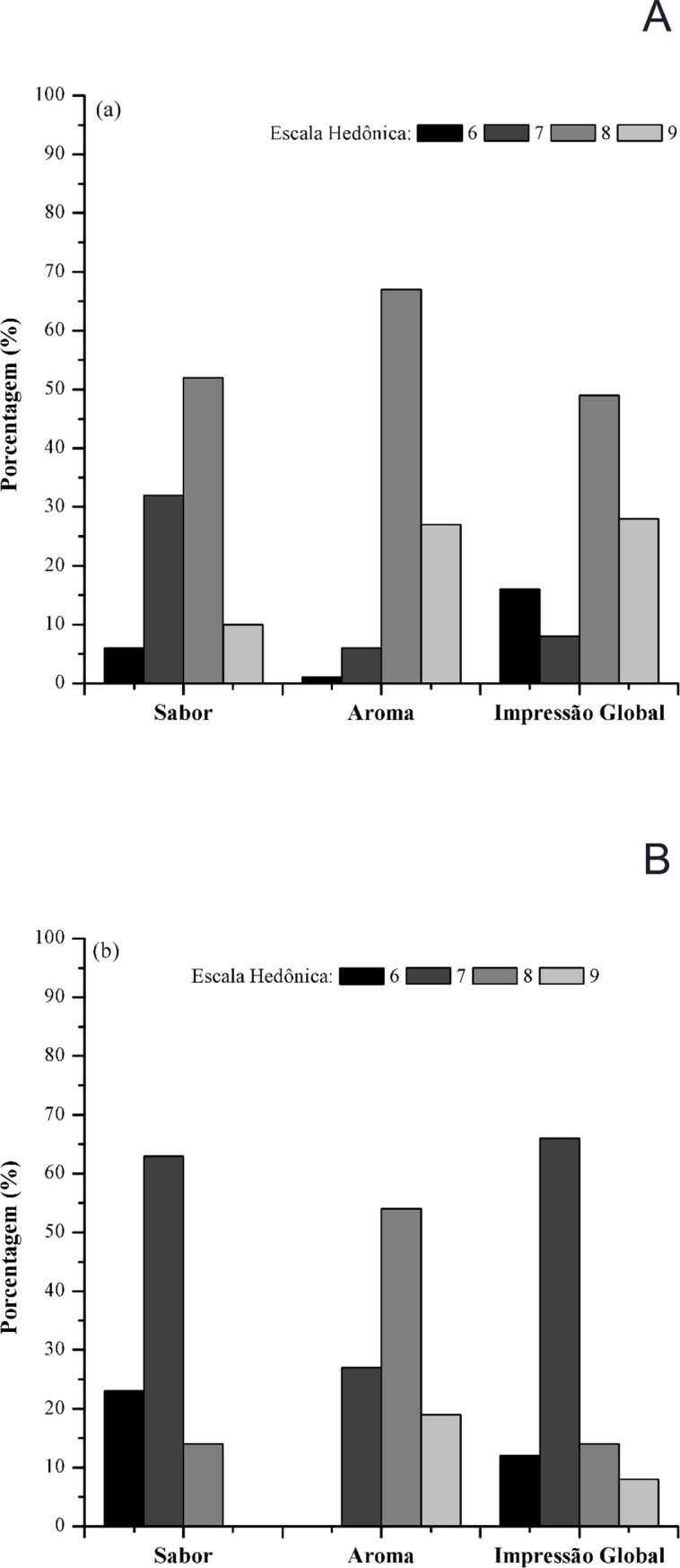
Acceptance tests of beer samples produced with 30% cocoa pulp as an adjunct and the yeasts SC52 (a) and S-04 (b).

Based on the evaluation of the tasters, there was a significant difference between the samples (p<0.05) in relation to the attributes. The samples were placed between the hedonic terms “like very much” and “like slightly”. The attribute aroma was the one with the highest marks, with 8.1 and 7.8 for both products. Regarding taste, the tasters attributed lower marks, with 6.88 for “like slightly” and 7.63 for “like moderately” for the beers produced with the yeasts S-04 and SC52, respectively. Based on the results of the acceptance test for the attribute overall impression, 49% of the tasters marked 8 for “like very much” and 28% 9 for “like extremely” for the beer produced with the yeast SC52; 66% of the tasters rated the beer produced with the commercial yeast a 7 for “like moderately” and 14% rated 8 for “like very much” (Fig 6). According to the ratings obtained, both beer samples were accepted, though the beer produced using SC52 had higher marks, indicating higher sensorial acceptability by the tasters.

**Fig 7:**
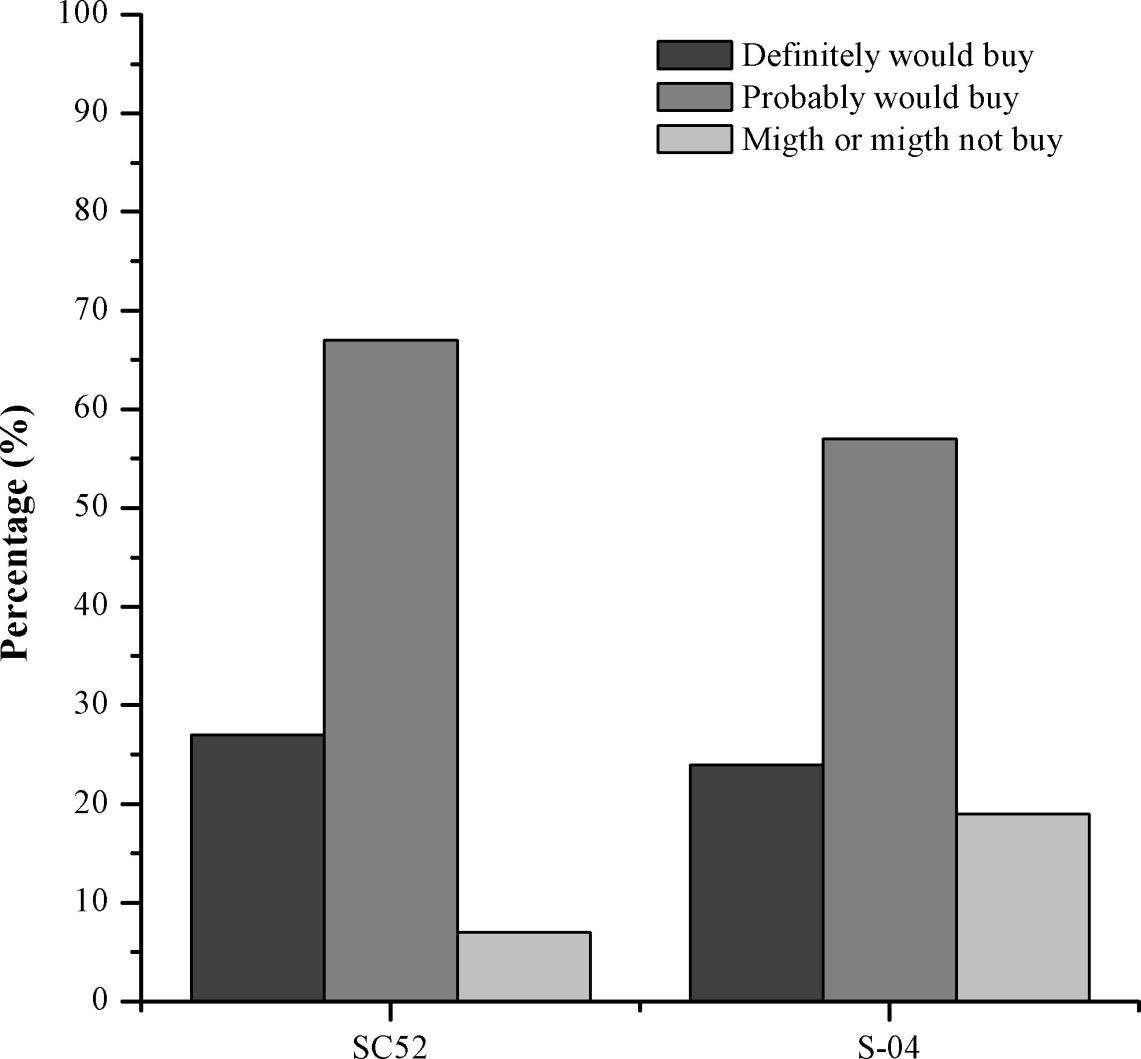
Intention-to-buy test for beers produced with 30% cocoa pulp as an adjunct and yeasts SC52 and S-04.

The same tasters were also used for the intention-to-buy test, which was aimed to complement the sensorial analysis. As shown in Fig 7, most tasters (60%) indicated that they would probably buy the beer produced using the yeast SC52, with 57% buying the beer produced with the commercial yeast; 27% and 24% indicated that they would certainly buy the beers produced with yeasts SC52 and S-04, respectively. Among the tasters, only 7% and 19% indicated that they were in doubt regarding whether they would buy the beer produced with yeasts SC52 and S-04, respectively. Therefore, it can be inferred that cocoa pulp as an adjunct conferred desirable characteristics to the product, especially the beer produced using yeast SC52.

## Discussion

The objective of this work was to evaluate the characteristics of beer produced with cocoa pulp, comparing the yeasts SC52 and S-04. The apparent extract consumption by SC52 was faster than that by S-04, with a consequent increase in ethanol production, as shown in Fig 2. This represents an increase in the brewing capacity, considering that the more complete the apparent attenuation, the more important it is to the process [7]. The use of an adjunct in the fermentation medium contributed to an increase in the concentration of fermentable sugars, especially glucose and fructose. According to Briggs et al. [1], Dietvorst et al. [29], and Gibson et al. [30], the main sugars in a 12°P all-wort malt are glucose and fructose (10%), maltose (45–65%) and maltotriose (15%).

Regarding the kinetics of sugar consumption, glucose and fructose were preferentially consumed by the yeasts, with glucose being completely consumed by yeast SC52. Other sugars, that is, maltose and maltotriose, were consumed in a higher quantity after almost all hexoses had been consumed. The higher consumption of sugars after 60 hours of fermentation coincided with the increase in ethanol production and remained for 96 hours, which determined the end of the fermentative process.

Compared to that using strain S-04, the beer produced with yeast SC52 showed a lower concentration of residual sugars. The data in the present study are similar to those reported by Nogueira et al. [31], who conducted an analysis of carbohydrates in pilsner beer produced using an adjunct. Previous research has indicated that the genes required to utilize maltose and maltotriose are repressed in the presence of high concentrations of glucose, fructose and sucrose [26]. A high concentration of residual sugars (maltose and maltotriose) was also observed in the beer when high concentrations of glucose were applied as an adjunct [32], a finding that was not observed in the present study with the yeast isolated from the fermentation of cachaça.

The production of ethanol started in the first hours of fermentation, initially showing low values and considerably increasing from 36 hours of fermentation, likely due to a reduction in the oxygen concentration. The levels of ethanol observed were 6.02% (v/v) and 5.95% (v/v) for strains SC52 and S-04, respectively. Compared with the study performed on a laboratory scale using 12°P all-malt wort, an increase in ethanol production was observed with the addition of cocoa pulp as an adjunct (data not shown).

The yeast SC52 resulted in higher productivity and yield in relation to S-04. The levels of ethanol in beers produced using starchy adjuncts analyzed by Bvochora and Zvauya [33] were 4.74% lower than those in the present study. According to Guido et al. [34], as most strains used in breweries show moderate tolerance to ethanol, high concentrations can halt the growth and multiplication of yeast cells in the brewing process. In our research, a higher increase in yeast biomass when using the SC52 strain was noted with adding cocoa pulp as an adjunct. A long latent phase was not observed before the start of ethanol production. Despite the increase in ethanol concentration, the yeasts SC52 and S-04 showed a high percentage of viability, between 93 and 90%, respectively, suggesting that the fermentation medium had not negatively affected their activity.

The production of glycerol, the most abundant of the secondary organic compounds of fermentation for both yeasts (SC52 and S-04), did vary greatly, being only slightly higher in the beer produced with the commercial yeast S-04. A tendency toward an increase in this metabolite throughout fermentation was observed, as shown in Fig 5. Accumulation of glycerol is a characteristic of the yeast stress response to hyperosmotic conditions and is also associated with maintenance of the cellular redox equilibrium, which is altered by the formation of organic acids, biomass and the presence of sulfite in the wort [35–36]. Methanol was not detected in the wort or in the final product.

The pH of beer is another parameter to follow during fermentation that determines quality. The pH values were 5.3 and 5.4 at the beginning of fermentation and 4.3 and 4.1 at the end for yeasts SC52 and S-04, respectively, in accordance with data in the literature. Under these conditions, beer becomes an unfavorable medium for the growth of most contaminating bacteria due to the low pH. Indeed, beer is a slightly acidic product, generally between 3.5 and 4.4, though there are some exceptions. pH variation is associated with the presence of organic acids.

Although these acids are normally derived from yeast metabolism, their concentrations in the final product also depend on the fermentation conditions [30]. The most important sensorial function of the organic acids in beer is to increase the acidity to a level that is pleasant to human taste. pH is a key factor that influences beer maturation, the stability of the taste and non-biological turbidity, and it has a significant effect on astringency and the perception of enhanced bitterness [1,37–38]. The beer produced with yeast SC52 exhibited a superior bitterness to that produced with strain S-04.

Bitterness is an attribute of quality and a typical characteristic of beer. It is almost entirely derived from the iso-α-acids of hops. The higher the IBU—or the more hops used—the more bitter the beer will be because the main component responsible for beer bitterness is the hops. However, during beer fermentation and maturation, the perceived intensity can be repressed by the formation of other compounds responsible for the flavor [1,39]. Color among the main characteristics of beer because it is an aspect of immediate perception and can potentially interfere with acceptance by the consumer. Regarding color, beers are considered light, as the color intensity is below 20 EBC units [40–42], and can be influenced by the addition of cocoa pulp. The color of beer is highly dependent on the raw materials used to produce the wort and is commonly related to the melanin and caramel present in malt [1,10,43].

In this study, the addition of cocoa pulp to the fermentation medium and the use of yeast isolated from the spontaneous fermentation of cachaça may have contributed to the increased production of esters, which are among the most important aromatic compounds in alcoholic beverages and are formed in low concentrations during alcoholic fermentation [44–46]. Of the volatile compounds present in beer, esters are a very important group with regard to the flavor composition. The most important esters that confer active flavors in beer were produced in significant quantities in the beers produced using cocoa pulp, with higher concentrations for strain SC52.

Normally, during the production of beer, several esters are present in concentrations close to the detection limit by the organoleptic threshold. This means that small changes in their concentrations can significantly alter the flavor of the beer [11,47–48]. In the present study, the concentrations of acetate and ethyl esters were higher in the beer produced with the S-04 yeast. Except for the ethyl acetate, which was found in lower quantity, the concentrations were also superior to the results of Hilaral et al. [49], who studied the aroma profile of different beers with various nutritional supplements and using the same strain. Ethyl acetate greatly influences the aroma and flavor of beer [38,50–51]. These data show that the composition of the fermentation medium and the temperature and type of yeast used are factors that interfere with the production of the compounds necessary for the beer aroma—in this case, esters.

The addition of adjuncts in beer formulations has contributed to the brewing of products with distinct characteristics. In addition to contributing to the fermentation performance, the use of cocoa pulp resulted in a product with good sensorial acceptance, with aroma being the most positively mentioned attribute by the tasters, corroborating the data on esters in beer. The beer produced with yeast SC52 received higher marks related to aroma, taste and overall impression, with higher concentrations of esters, which are responsible for the flavor of beer. Along with esters, it is important to outline that the quality of the final product depends on the conditions (e.g., pH and temperature) and other compounds that are formed during fermentation [52–56]. Based on the acceptance test, the beer produced using the yeast isolated from spontaneous fermentation of cachaça had higher acceptability.

## Conclusion

This study showed that cocoa addition at 30% to wort increased the initial concentration of fermentable sugars, favoring the fermentative performance of yeasts SC52 and S-04, with an increase in volumetric productivity and yield of ethanol.

The strain SC52, isolated from spontaneous fermentation of cachaça, showed a better fermentative performance than the commercial yeast S-04, with a higher consumption of substrate, ethanol production and formation of main beer esters (ethyl acetate, isoamyl acetate, isobutyl acetate, phenyl ethyl acetate, ethyl caproate and ethyl caprylate).

Our sensorial analysis revealed a good acceptance of the products, with the beer produced using yeast SC52 showing higher acceptability in relation to the attributes evaluated.

Thus, addition of cocoa pulp in beer production contributed to obtaining a product with more acceptable sensorial characteristics compared with beer produced using a commercial strain.

## Supporting information

S1 Fig Sensory evaluation. (DOC)
