# Supplementary material for: Cocoa pulp in beer production: Applicability and fermentative process performance
Source: PLoS One. 2017 Apr 18;12(4):e0175677. doi: 10.1371/journal.pone.0175677 (PMC5395165; doi:10.1371/journal.pone.0175677)
Supplement: S1 Fig — (DOC) [file pone.0175677.s001.doc]

| 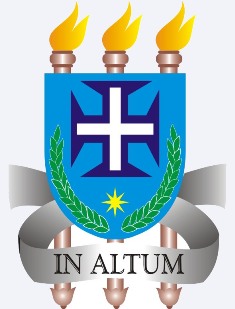 | NAME:  DATE:  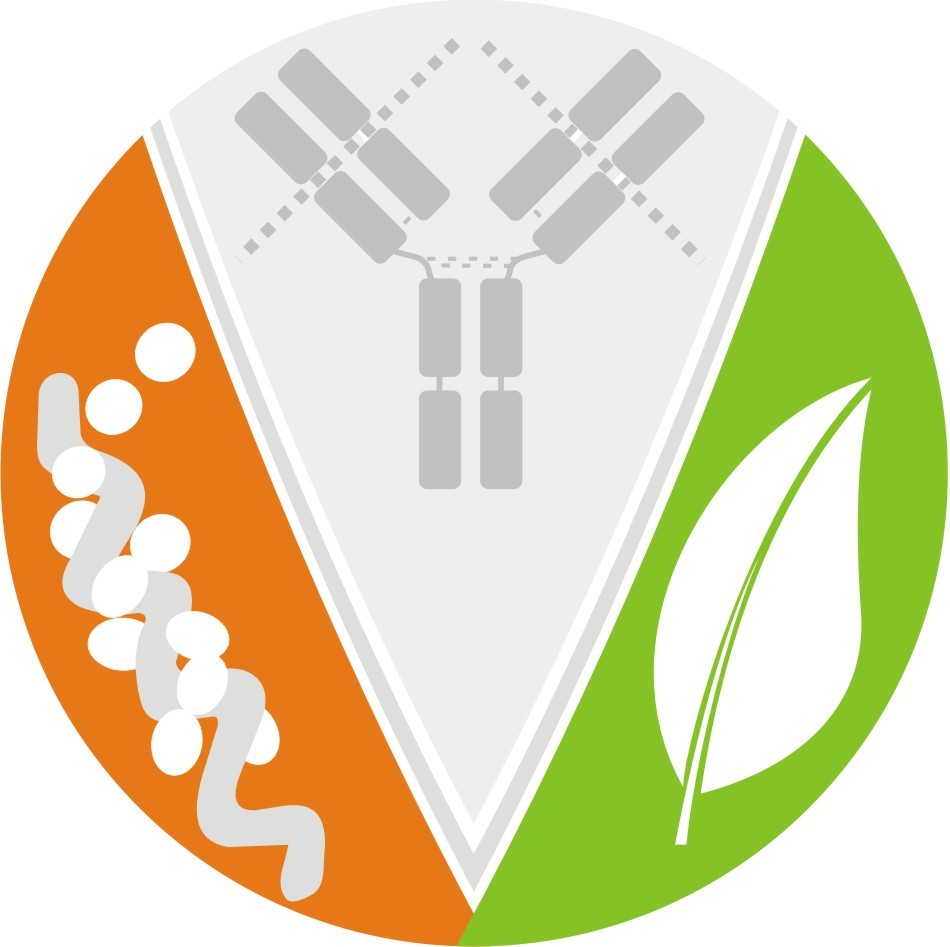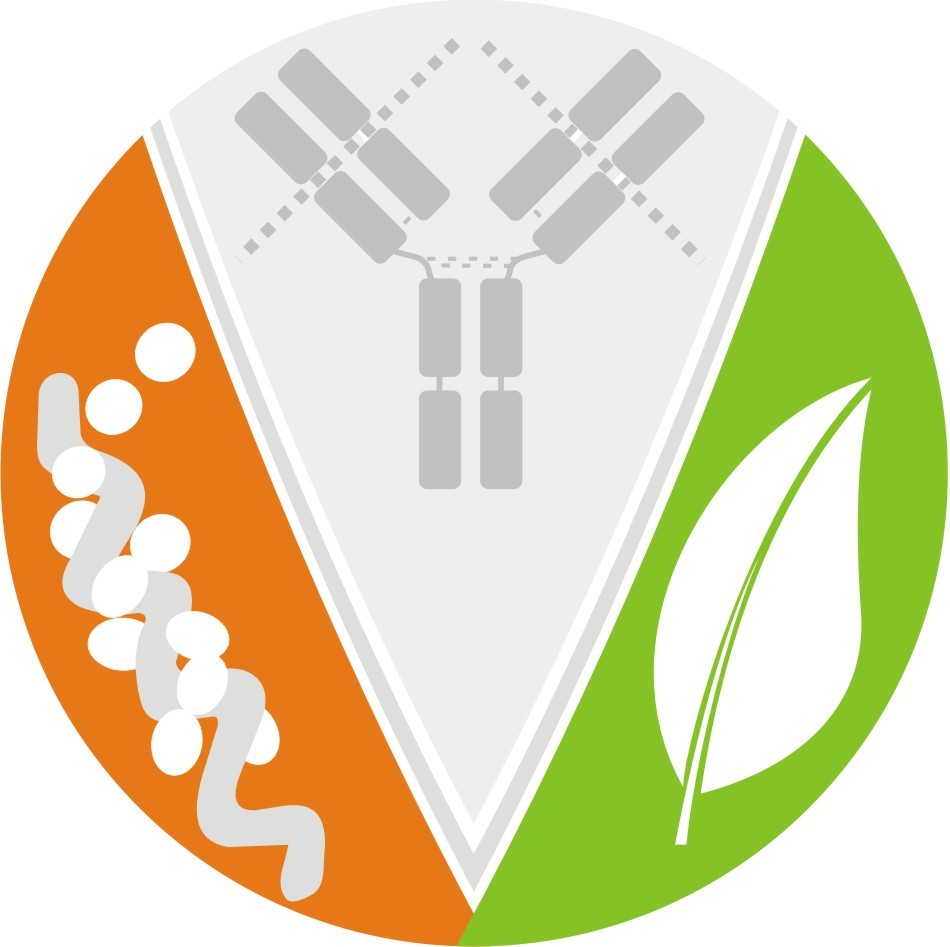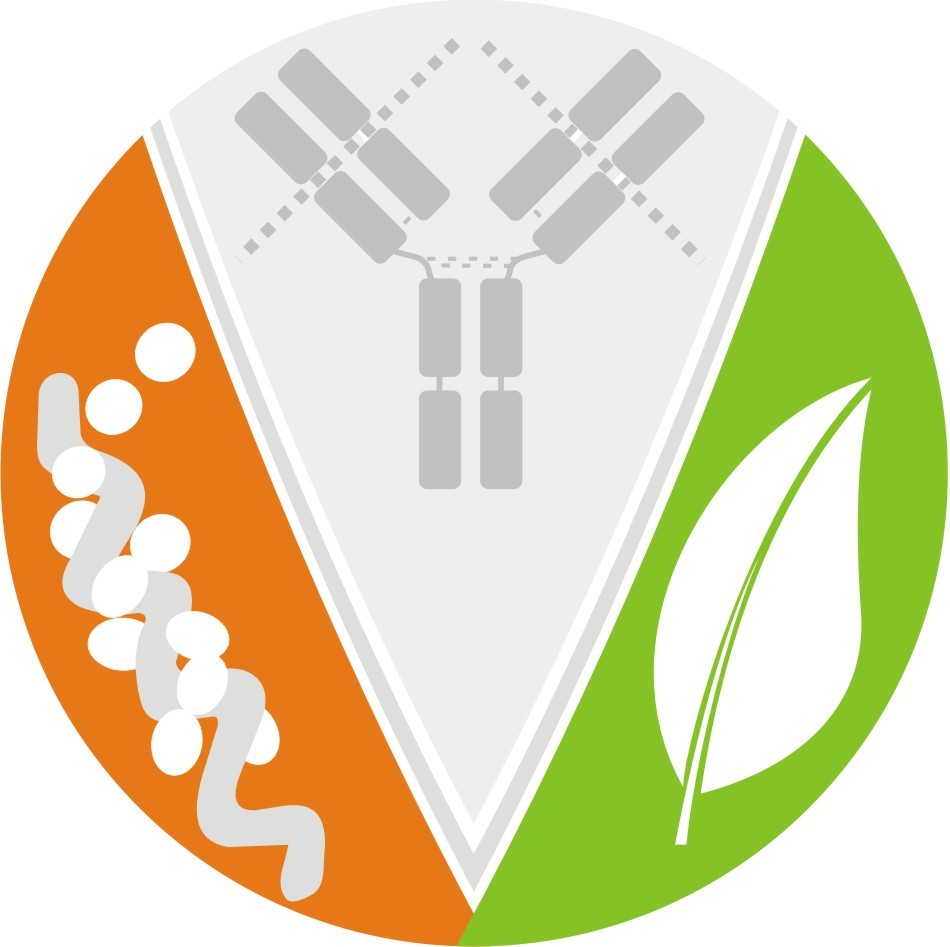 | | |
| --- | --- | --- | --- |
| You are receiving codified sample of beer. Please, taste the beer sample and indicate how much you liked or disliked the product regarding aroma, taste and overall impression. | | | |
| Hedonic scale:    9 - Like extremely.  8 - Like very much.  7 - Like moderately.  6 - Like slightly.  5 – Neither like nor dislike.  4 - Dislike slightly.  3 - Dislike moderately.  2 - Dislike very much.  1 - Dislike extremely. | | Sample | Number |
|  |  | Aroma  Taste  Overall impression | ___________  ___________  ___________ |
|  | | | |
| If these samples of beers were available on the market, what would be your purchase intention? | | | |
| Alternative:    (A) – Definitely would buy.  (B) – Probably would buy.  (C) – Might or might not buy.  (D) - Probably would not buy  (E) - Definitely would not buy. | | Alternative | |
|  |  | ___________ | |
| Comments: | | | |

S1 Figure**.** Sensory evaluation sheet
